# Evaluation of Genetic Diversity and Identification of Cultivars in Spray-Type Chrysanthemum Based on SSR Markers

**DOI:** 10.3390/genes16010081

**Published:** 2025-01-13

**Authors:** Manjulatha Mekapogu, So-Hyeon Lim, Youn-Jung Choi, Su-Young Lee, Jae-A Jung

**Affiliations:** Floriculture Research Division, National Institute of Horticultural & Herbal Science, Rural Development Administration, Wanju 55365, Republic of Korea

**Keywords:** chrysanthemum, cultivar identification, genetic diversity, molecular markers, ornamental plants, polymorphism, simple sequence repeat (SSR) markers

## Abstract

Background/Objectives: Chrysanthemum (*Chrysanthemum morifolium*), a key ornamental and medicinal plant, presents challenges in cultivar identification due to high phenotypic similarity and environmental influences. This study assessed the genetic diversity and discrimination of 126 spray-type chrysanthemum cultivars. Methods: About twenty-three simple sequence repeat (SSR) markers were screened for the discrimination of 126 cultivars, among which six SSR markers showed polymorphic fragments. Results: Results showed high polymorphism across six markers, with an average of 3.8 alleles per locus and a mean polymorphism information content (PIC) of 0.52, indicating strong discriminatory efficiency. The average observed heterozygosity (Ho) was 0.72, reflecting significant genetic diversity within the cultivars. Cluster analysis using the unweighted pair group method with arithmetic mean (UPGMA) grouped the cultivars into seven clusters, correlating well with the PCA. Bayesian population structure analysis suggested two primary genetic subpopulations. Conclusions: These findings confirm SSR markers as an effective tool for the genetic characterization and precise discrimination of spray type chrysanthemum cultivars, offering significant applications in breeding, cultivar registration, and germplasm conservation. The SSR marker-based approach thus provides a reliable and efficient strategy to enhance the management and commercialization of diverse chrysanthemum germplasm collections.

## 1. Introduction

Chrysanthemum *(C.morifolium),* belonging to the Asteraceae family, is a perennial flowering plant and a commercially influential ornamental crop. It is one of the top four most popular cut flowers after the rose, accounting for more than 30% of global cut flower production and making a substantial contribution to the international floral market. Chrysanthemum species also have medicinal and cosmetic value due to their antipyretic, antioxidant, and anti-inflammatory properties [1]. In addition to cut flowers, chrysanthemums are extensively used as potted ornamental plants, garden cover, and in landscaping [2]. The rich diversity in chrysanthemum’s floral colors, shapes, and sizes offers high aesthetic appeal and substantial economic significance. Ploidy levels in chrysanthemum species range from diploid to decaploid, with cultivated chrysanthemum exhibiting hexaploidy, comprising a stable configuration of 2n = 6x = 54 [3]. This complex genome and allohexaploid nature are the primary reasons for the exceptional diversity in both floral and architectural phenotypes [4].

The propagation of chrysanthemums by vegetative cuttings and plenty of inherent genetic variation allows easier genetic manipulations, resulting in diverse phenotypic variations [5]. This genome complexity allows for substantial possibilities for morphological variation even among closely related genotypes, leading to the commercialization of many cultivars annually, contributing to the multi-billion-dollar floral industry. Although hundreds of cultivars are available, the annual rise in global demand for chrysanthemum cut flowers drives breeders to develop novel cultivars. However, most cultivars share similar flower phenotypes, posing challenges for morphological differentiation [6]. Widely used traditional morphological identification methods are often limited by environmental influences and high morphological similarity among cultivars, leading to poor identification and classification on the market [7]. Additionally, the multigenic nature of morphological features and their dependency on specific growth phases, influenced by environmental conditions, hinder accurate cultivar characterization [8]. The identification and discrimination of varieties are vital for breeding, seed production, selection, registration, market trade, and inspection [9]. The identical floral phenotype among many commercialized cultivars, along with numerous synonyms for local varieties, results in incorrect labeling, posing significant challenges in cultivar assignment. Therefore, an efficient method is essential for accurate variety discrimination, precise market labeling, and securing breeders’ intellectual property rights.

Since phenotypic approaches are imprecise, labor-intensive, and less informative, molecular markers offer a rapid and cost-effective alternative for variety identification [10]. DNA fingerprinting methods provide promising applications for breeders and growers in cultivar identification, helping to avoid the problems of propagating or selling cultivars without permission or incorrect labels. Molecular marker-based identification is rapid compared to traditional approaches, which require growing questionable plants until the flowering stage [11]. Although traditional methods continue to be used for distinctiveness, uniformity, and stability (DUS) testing in the registration of new cultivars and for granting breeders intellectual property rights (IPR), these methods are less effective when rapid results are required and when large numbers of cultivars need to be distinguished. For decades, molecular methods and DNA fingerprinting have been widely applied for genotype and cultivar identification across various crops, enabling gene patenting, variety registration, and the detection of biopiracy and infringement of breeders’ intellectual property rights [12].

Molecular marker-based methods are recommended for the DUS testing of varieties with limited genetic diversity and are widely endorsed for the identification, certification, and protection of new cultivars by the International Union for the Protection of New Varieties of Plants (UPOV 2010) [13,14]. A wide variety of molecular markers have been previously applied in genetic studies of cultivated chrysanthemums [15,16,17,18]. Simple Sequence Repeat (SSR) markers have the advantages of co-dominance, abundance, their multi-allelic nature, high precision, and ease of scoring compared to other dominant markers, which are more difficult to reproduce [19]. SSRs are highly polymorphic genetic markers used in cultivar identification, pedigree reconstruction, and genetic mapping [20]. Like other crops, large numbers of new chrysanthemum cultivars are commercialized annually to meet global demand. Chrysanthemums are categorized as standard or spray type, with standard types exhibiting a single large bloom per stem, while spray types have multiple smaller blooms per stem [21,22]. Spray-type chrysanthemums, with a higher number of flowers displaying a diverse range of colors, shapes, and sizes, are highly desirable in the cut flower market. Several studies have successfully employed SSR markers for genetic diversity analysis and the identification of cultivars across various chrysanthemum types, including both spray and standard varieties [23,24,25,26,27,28].

Hence, this study aims to assess the effectiveness and informative capacity of SSR markers to characterize spray-type chrysanthemum cultivars. We examined the genetic characteristics of a diverse collection of these cultivars, evaluating the informativeness and utility of SSR markers for assessing genetic diversity.

## 2. Materials and Methods

### 2.1. Plant Material

A total of 126 spray chrysanthemum cultivars, including both Korean and foreign varieties, were used in this study. Rooting was induced from the plant cuttings of chrysanthemum cultivars and the plantlets with roots were raised in the pots filled with a soil and peat moss mixture and maintained in the natural greenhouse conditions. One-month-old plantlets were then transplanted to the artificial soil bed with a 15 × 15 cm density. The soil bed constituted perlite and peat moss in a 1:1 ratio. A randomized complete block design was employed in each line with five replications for each cultivar in two separate beds. Irrigation, fertilization, and other horticultural practices were followed through the cultivation period. The daily photoperiod was 14 h to 16 h long, with a light intensity of 100 µmol photons m^−2^ s^−1^. A temperature and relative humidity of 22 ± 5 °C and 70–75%, respectively, were maintained constantly in the green house. SSR analysis was performed in ten plants from each cultivar.

### 2.2. Genomic DNA Extraction

Fully expanded, young, and healthy leaves from each cultivar were collected, frozen in liquid nitrogen, and stored. Genomic DNA was extracted from the leaves using a DNeasy plant mini kit (Qiagen, Hilden, Germany). DNA quantity and quality were checked using the Quick Drop (Molecular Devices, San Jose, CA, USA). The samples were standardized to 25 ng µL^−1^ by diluting the DNA and used for SSR analysis.

### 2.3. SSR Amplification and Evaluation Using ABI Genetic Analyzer

The differentiation of the 126 chrysanthemum cultivars employed 23 SSR markers. Highly polymorphic primer pairs were obtained from the previous studies and after the primary screening of the twenty-three markers, six polymorphic SSR markers were selected to be used for the characterization of the selected cultivars in this study [25,26]. Details of the primers are presented in Table 1. The M13 tailing method was then applied for the labelling of PCR products [29]. The PCR amplification of each SSR marker was carried out by amplifying 25 ng of DNA in an independent reaction of 25 µL PCR reaction volume comprising 8 pmol (5′ FAM labelled) of primer pairs, 0.4 mM of dNTPs, and 0.3U of Taq DNA polymerase combined with 1X Taq buffer, making up the total volume to 25 µL with sterile water. The PCR reaction was executed in the thermocycler (Veriti, Applied Biosystems, Waltham, MA, USA). Following the initial denaturation at 94 °C for 5 min, 35 cycles of denaturation at 94 °C for 60 s, annealing at 58 °C for 30 s, and extension at 72 °C for 45 s were employed and ended with a 30 min final extension at 72 °C. The resulting PCR product (2 µL) was separated on 2.5% agarose gel. Clear and scorable amplification patterns were visualized by DNA Loading STAR (Dyne Bio, Seongnam, South Korea). The PCR reaction was repeated thrice to confirm its reproducibility. Further genetic analysis was performed by mixing each of the PCR products (1 µL) with Hi-Di formamide (10 µL) and GeneScan-500 ROX internal size standard (0.12 µL) per well. This mixture was further analyzed on an ABI PRISM 3100XL Genetic Analyzer (Applied Biosystems, Waltham, MA, USA). The resulting PCR fragment sizes, representing the corresponding SSR loci, were read using GeneMapper (version3.7) software. The data derived from the ABI genetic analyzer was further used for the genetic diversity analysis of the 126 chrysanthemum cultivars.

### 2.4. Data Analysis

Microsatellite allele parameters, such as genetic diversity metrics, heterozygosity, allele counts, allele frequency, and polymorphism information content (PIC), were analyzed using PowerMarker v3.25 software [30]. The amplified SSR fragments analyzed were scored as 1 for presence and 0 for absence. Genetic similarity clustering was performed using the unweighted pair group method of arithmetic averages (UPGMA), and a dendrogram was created using PAST v3.26 software with a bootstrap frequency of n = 100. Principal Component Analysis (PCA) was also conducted using PAST v3.26.

To identify clusters of genetically similar individuals, a population structure analysis was conducted using the Bayesian clustering approach in STRUCTURE v4.3.2 software (Stanford University, Stanford, CA, USA) [31,32]. This clustering method accounts for admixtures and correlated allele frequencies, providing insights into the origins and genetic admixtures of the cultivars. Genotypes were classified into subpopulations based on maximum membership probability [33]. Parameters were set with a burn-in period of 50,000 followed by 100,000 Markov Chain Monte Carlo simulations. Optimal subpopulation numbers (K values) were evaluated from 1 to 10 using an admixture model. Likelihood variation for each K value was assessed through ten independent runs, with the optimal K determined by LnP(K) and the second-order rate of change of likelihood (∆K) [31]. STRUCTURE Selector, a web-based tool, was used for calculations and graph construction [32].

## 3. Results

### 3.1. Assessing Genetic Variation of SSR Markers

**Figure 1:**
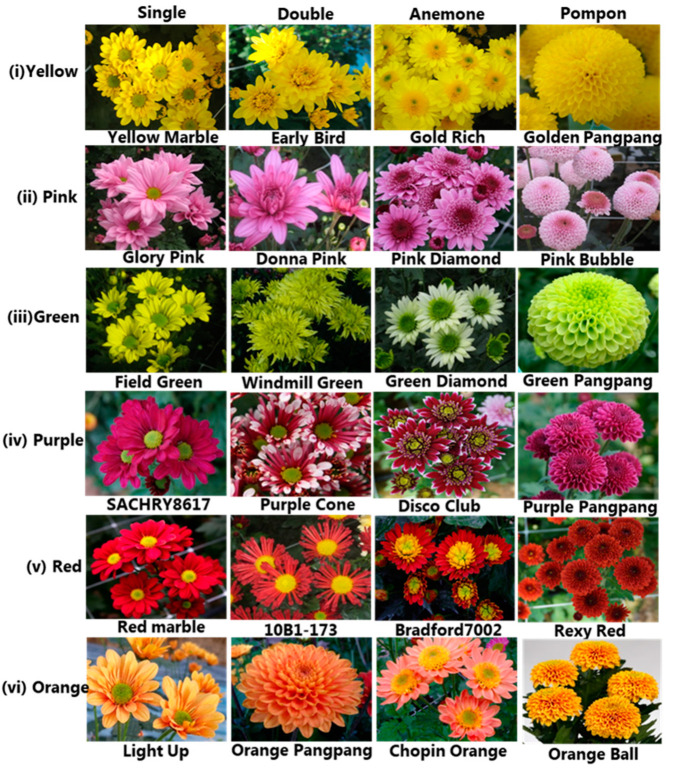
Spray type chrysanthemum cultivars representing various floral types and colors used in this study. (**i**) Yellow cultivars—Yellow Marble (Single (S)), Early Bird (Double (D)), Gold Rich (Anemone (A)), and Golden Pangpang (Pompon (P)); (**ii**) Pink cultivars—Glory Pink (S), Donna Pink (D), Pink Diamond (A), and Pink Bubble (P); (**iii**) Green Cultivars—Field Green (S), Windmill Green (D), Green Diamond (A), and Green Pangpang (P); (**iv**) Purple Cultivars—SACHRY8617 (S), Purple Cone (D), Disco Club (A), and Purple Pangpang (P); (**v**) Red cultivars—Red Marble (S), 10B1-173 (D), Bradford (A), and Rexy Red (P); and (**vi**) Orange Cultivars—Light Up (S), Orange Pangpang (D), Chopin Orange (A), and Orange Ball (P).

A total of 126 spray chrysanthemum cultivars, with diverse genetic backgrounds and exhibiting wide range of floral colors and floral types, were evaluated by applying SSR markers. A list of the tested cultivars, categorized by color and type, is shown in Table 2. Among these 126 cultivars, the majority had yellow flowers (37), followed by pink (29), green (23), purple (19), red (12), and orange (6). This collection included various flower types, such as single, double, semi-double, anemone, and pompon (Figure 1).

**Figure 2:**
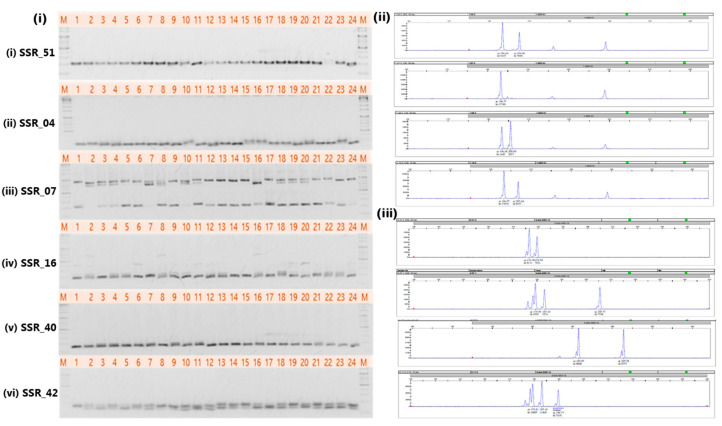
Representative images of (**i**) PCR amplicons of six SSR markers tested and separated on agarose gel; and (**ii** & **iii**) chromatograms of SSR_51 and SSR_16 in different samples by ABI genetic analyzer 3100xl.

Initially, twenty-three markers from the chrysanthemum SSR database were screened to identify the highly polymorphic SSR markers, resulting in the detection of ten SSR markers. After the further screening of these ten markers, six pairs of microsatellite primers were observed to be highly polymorphic and were analyzed to assess their polymorphic efficiency among the 126 cultivars. All six SSR markers showed polymorphism across the chrysanthemum cultivars (Figure 2i). Representative results obtained for the loci SSR_51 and SSR_16 through the ABI genetic analyzer in different samples has been shown in Figure 2 (ii and iii). Genetic variation analysis showed that these six SSR markers generated 840 scorable bands representing 25 different alleles. The amplicon sizes of markers varied from 142 to 274 bp. The number of alleles per marker were either three (SSR_51, SSR_40), four (SSR_42), or five (SSR_4, SSR16), with a mean of 3.8 alleles per locus. The six SSR markers showed an average major allele frequency (M_AF_) of 0.48 per locus, with observed heterozygosity (HO) ranging from 0.60 to 0.79, with a mean of 0.72 (Table 3). The selected 126 cultivars showed an average gene diversity of 0.58, ranging from 0.39 to 0.70. Tested SSR markers exhibited Polymorphism Information Content (PIC) varying from a low of 0.30 in SSR_51 to a high of 0.63 in SSR_16, with a mean PIC of 0.52 (Table 3).

### 3.2. Genetic Relationship Assessment of the Chrysanthemum Genotypes

**Figure 3:**
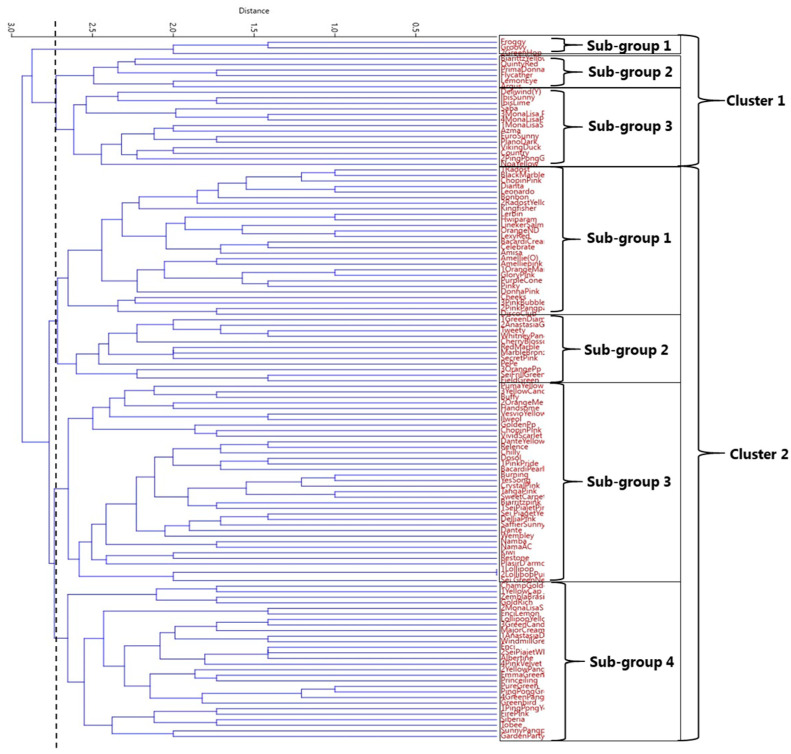
Dendrogram illustrating the classification of 126 spray chrysanthemum cultivars, established based on a UPGMA analysis using SSR markers. Various clusters are shown on the right side of the dendrogram. The scale at the top is the Euclidean distance.

The genetic relationships among the 126 spray-type chrysanthemum cultivars were evaluated by constructing a dendrogram based on the molecular profiles produced by the six SSR markers. A UPGMA cluster analysis was performed based on the Euclidean genetic distance values to construct a dendrogram. The Euclidean distance coefficient ranged from 0.10 to 3.00 across all the SSR markers. The dendrogram grouped all 126 cultivars into two major clusters, and at a distance of 2.75, cultivars were grouped into three and four subgroups in Cluster 1 and Cluster 2, respectively (Figure 3).

The clustering analysis grouped the 126 chrysanthemum cultivars into two major clusters with three and four sub-groups within each cluster. In Cluster 1, sub-group 1 contained three green, pompon-type cultivars, while sub-group 2 included six cultivars comprising two spoon types and one each of pompon, semi-double, single, and anemone types, with respective flower colors of yellow, red, pink, purple, and pink. Sub-group 3 included nine yellow cultivars (including four decorative, three single, and one each of anemone and pompon types), three pink cultivars (two anemone and one decorative), one purple (anemone), and one green (pompon). Sub-group 1 of Cluster 2 was a larger group containing 27 cultivars: 11 pink (five single, four pompon, and two decorative), six yellow (all single), three purple (two anemone, one single), three orange (two single, one semi-double), two red (one single, one pompon), and two green (anemone). Sub-group 2 comprised 12 cultivars, including five green (two single, one each of anemone, decorative, and pompon types), two single types in yellow and pink, and one pompon type each in purple, orange, and red. The sub-group 3 was the largest and included 36 cultivars, primarily pink (12), yellow (8), purple (5), red (5), green (3), and orange (3), spanning various floral forms. Finally, Cluster 7 consisted of 28 cultivars, predominantly yellow (11), followed by green (9), purple (2), and three each of purple and red cultivars.

**Figure 4:**
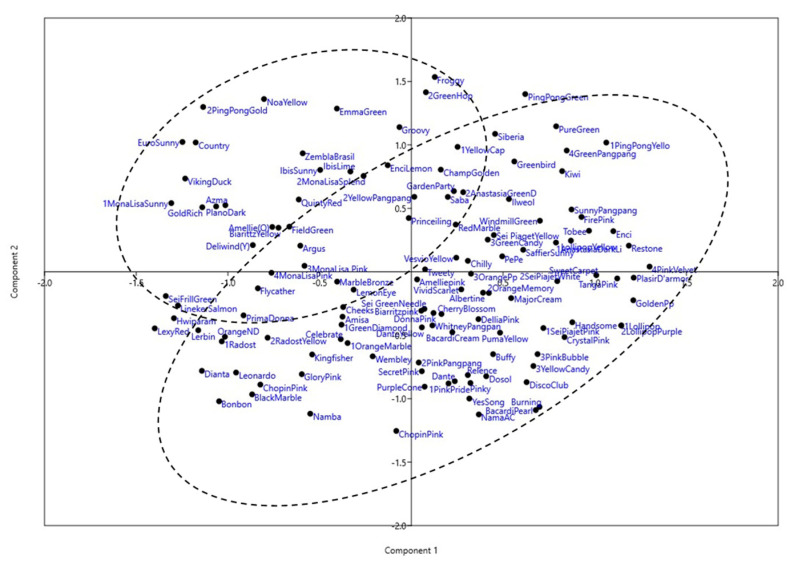
Plot depicting the principle component analysis (PCA) of 126 chrysanthemum cultivars based on SSR markers.

Certain cultivar groups showed close genetic relationships within clusters. For instance, two yellow decorative-type cultivars from the Ibis series, Ibis Sunny and Ibis Lime, grouped closely in sub-group 3. Similarly, three anemone-type Mona Lisa cultivars with pink, purple, and yellow flowers clustered together in sub-group 3. However, Mona Lisa Splendid, another cultivar in this series with yellow anemone-type flowers, was positioned distantly in sub-group 4, suggesting a different genetic background despite similar floral characteristics. Other examples include pink and yellow pompon cultivars Sei Piaget Pink and Sei Piaget Yellow, and the purple Namba and Namba AC cultivars with anemone and single flower types grouped in sub-group 3 of Cluster 2. Purple pompon cultivars Lollipop and Lollipop Purple showed a very close genetic relationship, indicating a shared genetic background. The Principal Component Analysis (PCA) of the SSR data supported the dendrogram findings, with cultivar groupings in the PCA plot aligning with the UPGMA cluster analysis (Figure 4).

### 3.3. Population Structure Analysis

**Figure 5:**
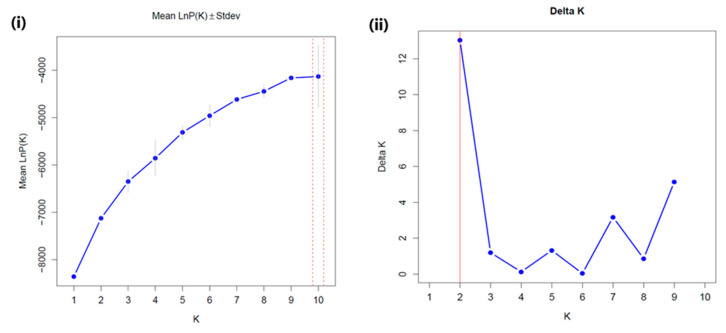
Graphical illustration of the assessment of the best subpopulation numbers according to the appropriate K value. (**i**) Mean of ∆K values representing 15 independent runs with K = 1 to K = 10 based on LnP(K) values. (**ii**) The mean of ∆K showed a peak at K = 2.

**Figure 6:**
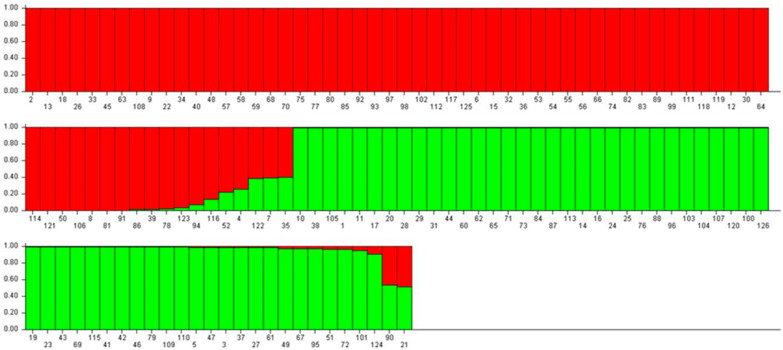
Genetic relatedness of individuals from 126 chrysanthemum cultivars, as analyzed by the STRUCTURE software (v4.3.2). Y-axis values represent membership coefficients to subpopulations, while X-axis values indicate the individual chrysanthemum cultivar codes of cultivars.

A population structure analysis was performed to assess genetic relationships and to identify subpopulations among the cultivars. The number of genetically distant subpopulations in the studied chrysanthemum cultivars were evaluated using the structure analysis. Individual cultivars were classified into relevant subpopulations based on the systematic analysis of the population structure using the Bayesian method in the Structure software (v4.3.2). In this method of clustering, individual genotypes are allocated to K clusters, where the Hardy–Weinberg law of equilibrium and linkage equilibrium are valid within the clusters, while being absent between the clusters [34]. In our study, the ∆K analysis revealed that the mean ∆K values were K = 2 among the 10 runs. Hence, K = 2 was the suitable value, suggesting that the studied population is subdivided into two subpopulations (Figure 5). Figure 6 displays admixture plots for K = 2. At the ideal K = 2, all of the 126 cultivars were categorized into two subpopulations. Group 1 comprised 59 cultivars and Group 2 included 49 cultivars, whereas 17 cultivars shared a mixed population ancestry.

## 4. Discussion

The annual commercialization of numerous chrysanthemum cultivars, combined with high morphological similarity, similar propagation methods, and shared genetic backgrounds, presents significant challenges in distinguishing cultivars. Furthermore, local nomenclature variations can complicate cultivar identification, which is essential for breeding, cultivar registration, breeder’s intellectual property rights, market introduction, and trade. Genetic characterization essential for the differentiation of germplasm can be effectively determined by molecular markers [34,35]. As SSR markers are advantageous over other markers in terms of high variability, co-dominance, cost-effective, precision and reproducibility, they have been widely used for genetic characterization studies. SSR markers have been used in constructing molecular maps and for intellectual property rights evaluations [36]. Various earlier studies have highlighted their effectiveness in distinguishing chrysanthemum genotypes [23,37,38].

Previous studies established the SSR database that can be used for the assessment of genetic relationships and the discrimination of cultivars in chrysanthemums [7,25,39]. For example, Shim et al. and Olejnik et al., in separate studies, used about 14 SSRs each to assess the genetic relationship among 147 and 97 chrysanthemum cultivars, respectively [7,25]. Recent studies have employed SSR markers exclusively for standard-type chrysanthemums, using different sets of SSRs to distinguish between cultivars [26,27,40,41]. In these studies, eight, twenty-six, six, and twelve SSR markers were successfully applied to differentiate fifty-six, thirty-six, eleven, and seven standard-type cultivars, respectively [26,27,40,41]. Additionally, a significant number of closely related white-colored chrysanthemum cultivars have been evaluated and genetically characterized using SSR marker sets [28].

In this study, we employed twenty-three SSR markers and, after the thorough screening of these markers, six SSRs were used to assess the genetic diversity of 126 chrysanthemum genotypes, representing various floral types and colors. Microsatellite parameters representing genetic diversity indicated the polymorphic nature of the six tested SSR markers (Table 3). The number of alleles produced by SSRs ranged from three to five per locus, with an average of 3.8 alleles per locus, which is similar to or higher than the previous reports of SSR markers in chrysanthemums, with an average of 5.6, 3.5, and 3.7 alleles per locus [26,28,41]. Variations in the number of alleles per locus can be affected by a number of factors including geographical origin, number of genotypes tested, and the different types of loci [42]. Observed heterozygosity (Ho) represents the genetic variability within the genotypes [43]. The average Ho in this study for six markers was 0.72, indicating a higher genetic variability in the 126 cultivars. This is comparable with findings from other chrysanthemum studies reporting Ho average of 0.88, 0.89 0.67, 0.81, and 0.75 [23,28,41,44,45]. Based on these results, the average Ho can vary depending on the number of markers used. The PIC, representing the degree of microsatellite polymorphism for the six SSR markers in the evaluated 126 cultivars, ranged from 0.30 to 0.65, with an average of 0.52, indicating a high degree of polymorphism in the SSR markers, as described by Botstein et al. [46]. In separate studies, Khaing et al. [44], Jo et al. [24], and Chang et al. [38] observed a higher PIC of 0.88 and a study by Kobeissi et al. [47] showed a PIC of 0.79. Slightly lower PICs of 0.50 and 0.53 were observed for 95 and 57 chrysanthemum cultivars, whereas a higher PIC of 0.9 was recorded for 32 chrysanthemum cultivars [18,28,48]. Thus, PIC demonstrates the discriminatory efficiency of SSR markers, inferring the genetic diversity of genotypes.

Genetic variations by SSR markers reveals the genetic relationship between the cultivars. In our study, tested SSR markers facilitated the genetic characterization of 126 cultivars and the UPGMA dendrogram constructed based on the SSR data clustered the cultivars into seven groups at a distance coefficient of 2.75 (Figure 3). Each cluster grouped genetically similar cultivars, such as the three green pompon cultivars in sub-group 1 of Cluster 1. Notably, two yellow decorative cultivars, Ibis Sunny and Ibis Lime, were closely related within the same cluster, as were three differently colored cultivars from the Mona Lisa anemone-type series (pink, purple, and yellow) in sub-group 3. In sub-group 1 of Cluster 2, two pompon-type cultivars, Sei Piaget Pink and Sei Piaget Yellow, showed close genetic similarity. Such genetic resemblance may result from the development of cultivar series with different floral colors through mutation breeding, which is aimed at generating new colors without altering other traits. For instance, Nagatomi et al. successfully produced six mutant chrysanthemum varieties with diverse floral colors from a single parent [49]. Additionally, cultivar pairs such as Namba and Namba AC, as well as Lollipop and Lollipop Purple in Cluster 6, exhibited near-identical genetic profiles, suggesting a shared genetic background. Previous research has similarly observed high genetic similarity between standard and spray-type chrysanthemums [26,27,28]. The efficiency of SSR markers for cultivar discrimination has been well demonstrated in recent studies. For example, 97 chrysanthemum cultivars were successfully distinguished based on different flower sizes using 14 SSR markers, with small-flowered cultivars displaying higher genetic diversity [7]. Another study analyzed 36 chrysanthemum genotypes using 26 polymorphic SSR markers, revealing high genetic diversity among the genotypes [41]. However, the lower resolution level with a weaker branch support among the clusters is a limitation, which could be due to the insufficient number of SSR markers used and hence, testing with a greater number of SSR loci could efficiently differentiate the cultivars with a stronger resolution.

Population structure analysis in plants reflects various genetic factors, including evolutionary history and gene flow within and between species [35]. In our study, the population structure analysis indicated that the most suitable K value for the 126 cultivars was two, grouping the cultivars into two genetic pools ( Figure 5; Figure 6). A mixed population ancestry was observed across clusters, with cultivars like Namba, Secret Pink, Fire Pink, Country, Lerbin, Biarrittz Pink, Noa Yellow, and Yellow Pangpang in Group 1 and Cheeks, Namba AC, Pink Pride, Hwiparam, Chilly, and Siberia in Group 2 showing notable levels of admixture. This pattern aligns with previous chrysanthemum studies, where population admixtures suggested genetic groupings of K = 2, K = 3, or K = 4 [7,41,48]. Mixed population structures in chrysanthemums are likely due to factors such as high heterozygosity, self-incompatibility, and the breeding and domestication history of the species [16,50].

## 5. Conclusions

In conclusion, this study provides a comprehensive genetic characterization of a diverse collection of 126 spray-type chrysanthemum cultivars, encompassing various floral types and colors. The results highlight the effectiveness of SSR markers in elucidating the genetic relationships among these cultivars, facilitating precise discrimination of individual genotypes within the collection. The SSR marker-based approach proves to be a valuable tool for distinguishing closely related genotypes and can be applied to the pedigree analysis, certification, and registration of chrysanthemum cultivars. Here, six SSR markers were found to be efficient in characterizing the larger number of cultivars in this study. However, the dendrogram differentiating the cultivars showed a weaker branch support, which is a limitation of the lower number of SSR markers applied. Hence, employing a greater number of markers in future studies would be more effective for the rigorous genetic characterization of closely related cultivars. These findings therefore establish a foundational platform for leveraging SSR markers in breeding programs, germplasm conservation, and accelerating selection processes through the genetic characterization of extensive genetic resource collections.

## Figures and Tables

**Table 1:** Details of the SSR primer pairs that were used to characterize 126 spray-type chrysanthemum cultivars.

No.	Marker	Primer Sequence		Repeat Motif
		Forward	Reverse	
1	SSR_48	TGAGATCATTCCCAACCTCC	CTAGCGTCCAAAGAATTGGC	(CCA)_5_
2	SSR_51	CCCCCTCTTCTTCTTCAACC	CAATAGAAAGCGCGTGACAA	(CCAA)_4_
3	SSR_52	AGTGACCCGAGCCAGATAGA	CCGACAAATCATTTCCGTCT	(ATA)_5_
4	SSR_127	TAACAAGGGGTTTCAGCGTC	TCAGGAAGAACAACCCAACC	(GGT)_5_
5	SSR_200	CCCAGAGAAGCGTGAGATTT	TCCCCTGCTACTACCACCAC	(GGT)_5_
6	SSR_222	AGCTAAAACAAACAAGCGGC	GCGTTAACTGTGTCGGTTGA	(ATC)_5_
7	SSR_330	CTGTTGAGCAGTTCAGGCAC	GTGTGATCGAGGCGATTTTT	(CAA)_5_
8	SSR_332	ACACCGAATAGGACGGACAG	TTTTCTGAAGTCCCGACCAC	(TGG)_5_
9	SSR_380	ACCAAAGGCAGCTCACAGTT	CCTCCCTCACTCATCTCTGC	(TGA)_5_
10	SSR_649	TCTTCCTCACACGCAAACAC	AGCTGCCACTCGCTATCACT	(ACC)_7_
11	SSR_706	CGATCACCATTCTTTTCCCA	CCGATAAGTTCGTCCTTGGT	(AG)_8_
12	SSR_728	TGGTTATGGGTGACCCTGAT	AAGAAAGTGCAGGCCAAGAA	(ATG)_5_
13	SSR_792	AGGAAGAAGATCGACACCCA	AAGTTCGGGTTTCCCCATAC	(TGA)_6_
14	SSR_863	CACAACCAGACAAGCCTTCA	ACTAACGGCGGTAGCTGAGA	(TC)_6_
15	SSR_4	ACAGTACACACACAGCCAACAC	GTAATGCTTCCGTCTGCATAGC	(CAA)_4_
16	SSR_7	AGGCCCAACTTTATTCACCC	CCAAATCCAATCTCCGGC	(GAG)_4_
17	SSR_9	CATTCACACTCACCTACACACC	CCTCCCTCTCTTACAATGTCAC	(TCA)_4_
18	SSR_16	ACAAGTAGTAGGAGGAGGAGGA	CAGTGTAGCCGGTACAGAAGA	(AGT)_5_
19	SSR_31	GGAAGCAAGTGTGTGGTTTC	ACCTCCCCATAGAATCTTGAGC	(TG)_9_
20	SSR_40	GACGGATTTTGAGCTTGGAG	GAACCAATAATCCCGACACC	(TA)_13_
21	SSR_42	CAAAGTACTACCAAACGCGG	GTAACATTGAGGGTGTAGCAGC	(CA)_6_
22	SSR_45	AAACAGCCTGACCCAATCTC	GTCATCATCCAACCACCAAC	(ATT)_6_
23	SSR_50	GATGGTGAGACATTGCGTCT	GCTCAAGGATTATGGACACTGG	(TTAT)_4_

**Table 2:** List of the 126 tested cultivars in this study, categorized by their flower color and type.

	Single	Pompon	Anemone	Decorative
Yellow	Biarittz Yellow, Fly Cather, Azma, Viking Duck, Noa Yellow, Lerbin, Hwiparam, Linekar Salmon, Bacardi Cream, Celebrate, Amisa, Tweety, Marble Bronze, Vesvio Yellow, Enci Lemon, and Major Cream.	Ping Pong Golden, Yellow Candy, Sei Piaget Yellow, Restone, Yellow Cap, Lollipop Yellow, Yellow Pangpang, and Ping Pong Yellow.	Mona Lisa Sunny, Radost Yellow, Puma Yellow, Ilweol, Gold Rich, Mona Lisa Splendid, and Garden Party.	Deliwind, Ibis Sunny, Ibis Lime, Euro Sunny, Dante Yellow, Saffier Sunny, Champane Golden, and Zembla Brazil.
Pink	Leonardo, Kingfisher, Amellie Pink, Glory Pink, Pinky, Cherry Blossom, Secret Pink, Pink Pride, Bacardi Pearl, Yes Song, Tanga Pink, Biarittz Pink, Dellia Pink, and Plasir D’amour.	Bonbon, Cheeks, Pink Bubble, Pink Pangpang, Sweet Carpet, Sei Piajet Pink, and Sei Piajet White.	Argus, Saba, Mona Lisa Pink, and Chopin Pink.	Plano Dark, Dianta, Donna Pink, Crystal Pink, Dante, Pink Velvet, Prima Donna, and Enci.
Green	Sei Frill Green, Field Green, Buffy, Sei Green Needle, and Emma Green.	Froggy, Groovy, Green Hop, Country, Whitney Pangpang, Kiwi, Green Candy, Pure Green, Ping Pong Green, Green Pangpang, Green Bird, and Siberia.	Radost and Green Diamond.	Antasia Green Dark, Antasia Dark Lime, and Windmill Green.
Purple	Lemon Eye, Purple Cone, Handsome, Namba AC, and Fire Pink.	Pepe, Lollipop, and Lollipop Purple.	Mona Lisa Purple, Chopin Purple, Disco Club, Namba, and Albertine.	
Red	Black Marble, Red Marble, Relence, Chilly, Dosol, Burning, Wembley, and Tobee.	Quinty Red, Lexy Red, and Sunny Pangpang.		Princeiling
Orange	Amellie (O), Orange Marble, and Vivid Scarlet.	Orange Pangpang and Golden Pangpang.		Orange ND and Orange Memory.

**Table 3:** Microsatellite allele metrics constituting allele number, gene diversity, M_AF_, H_O_, and polymorphism information content in 126 spray-type chrysanthemum cultivars based on SSR markers.

S.No.	Marker	Repeat Motiff	Allele Size (bp)	No. of Alleles	Gene Diversity	^¶^ M_AF_	* Ho	^§^ PIC
1	SSR_51	(CCAA)4	197-252	3	0.39	0.62	0.76	0.30
2	KChSSR_4	(CAA)4	248-274	5	0.70	0.38	0.79	0.65
3	KChSSR_7	(GAG)4	240-267	3	0.62	0.44	0.60	0.57
4	KChSSR_16	(AGT)5	185-194	5	0.68	0.40	0.77	0.63
5	KChSSR_40	(TA)13	146-152	3	0.58	0.48	0.66	0.49
6	KChSSR_42	(CA)6	142-170	4	0.53	0.55	0.75	0.46
				3.83	0.58	0.48	0.72	0.52

^¶^ Major allele frequency (M_AF_); * observed heterozygosity (H_O_); and ^§^ polymorphism information content.

## Data Availability

All datasets generated and analyzed in the current study are available from the corresponding author upon request.
